# Some practical guidelines for UV imaging in the protein crystallization laboratory

**DOI:** 10.1107/S1744309112048634

**Published:** 2013-01-26

**Authors:** Sebastien Desbois, Shane A. Seabrook, Janet Newman

**Affiliations:** a CSIRO Materials, Science and Engineering, 343 Royal Parade, Parkville, VIC 3052, Australia

**Keywords:** UV imaging

## Abstract

High-throughput imaging of protein crystallization experiments with ultraviolet (UV) light has recently become commercially available and can enable crystallographers to differentiate between crystals of protein and those of salt, as the visualization of protein crystals is based on intrinsic tryptophan fluorescence. Unfortunately, UV imaging is not a panacea, as some protein crystals will not fluoresce under UV excitation and some salt crystals are UV-fluorescently active. As a new technology, there is little experience within the general community on how to use this technology effectively and what caveats to look out for. Here, an attempt is made to identify some of the common problems that may arise using UV-imaging technology by examining test proteins, common crystallization reagents and a range of proteins by assessing their UV–Vis absorbance spectra. Some pointers are offered as to which systems may not be appropriate for this methodology.

## Abstract 2

The use of UV imaging as a means of locating protein crystals is a fairly new tool, however not suitable for all protein crystallization trials. Practical examples of the strengths and some of the pitfalls of the technology are presented.

## 1. Introduction   

The primary technique used to generate three-dimensional atomic-level structural information from biomacromolecules, X-ray crystallography, requires the production of suitable crystals for diffraction analysis. The production of crystals is laborious and, even more discouragingly, highly stochastic (Newman *et al.*, 2012 ▶). Literally millions of crystallization trials are set up annually, and currently the best way to detect crystal growth is human inspection; automated image analysis at this moment is prohibitively complex to initiate. The rate of false negatives in identifying protein crystals using the human eye reaches 20% (Cumbaa & Jurisica, 2010 ▶), and given a success rate of less than 1% overall (Newman *et al.*, 2012 ▶), this rate of false negatives becomes intolerable. A false negative may be a result of the loose arrangement of protein molecules in a crystal lattice (Matthews, 1968 ▶), which incorporates large solvent channels. The solvent channels can create protein crystals with a refractive index close to that of the mother liquor in which they grew, rendering them invisible under visible light. False positives, where objects in crystallization trials are interpreted as protein crystals despite not being so, are less problematic, but many a crystallographer has wasted significant resources following such spurious leads.

UV-light illumination claims to reduce the rates of both false positives and false negatives. It is a relatively new technique for the identification of protein crystals; it is based on the assumption that the local protein concentration is greatest when crystalline, and thus crystals will ‘shine’ more brightly than the surrounding solution. This technique requires specialized hardware and consumables (Dierks *et al.*, 2010 ▶; Gill, 2010 ▶) such as a UV-equipped microscope and low-UV-absorbing plates and seals. Commercial UV imaging commonly uses UV light at 295 nm; consequentially, the resulting UV images result from only the intrinsic tryptophan fluorescence of the protein. Tryptophan absorbs light at a wavelength of 290 ± 5 nm, with a solvatochromic fluorescent emission of 320–350 nm (Permyakov, 2012 ▶). Other technologies used to identify positive results in crystallization have included dye-based fluorescence systems (Pusey, 2011 ▶) and second-order nonlinear imaging of chiral crystals (SONICC; Kissick *et al.*, 2011 ▶).

All amino acids with an aromatic side chain are capable of absorbing UV light; however, only tryptophan displays a sufficiently high quantum efficiency at 290 nm to be useful as a fluorescent probe. Likewise, disulfide bonds are capable of absorbing UV light, but do so at 260 nm and then only weakly (Schmid, 2001 ▶). Tryptophan makes up an average of 1.09% of the residues in proteins (Gilis *et al.*, 2001 ▶), and theoretically this level is high enough to provide the intrinsic fluorescence required for UV imaging. Based on these concepts, a crystal which is observed under visible-light inspection but not under UV illumination is very likely to be a salt crystal, and crystals can be distinguished in UV images even if the visible-light image is badly lit or out of focus or if there is a confounding background of precipitated protein. It has been shown that variation in the pH can affect fluorescence in general; however, tryptophan fluorescence has been reported to be pH-insensitive (Chen, 1973 ▶). Unfortunately, there are a number of known physical phenomena that will affect protein fluorescence or create misleading results: the experimental setup may be inappropriate, the protein precipitate may glow too brightly to allow crystals to be distinguished, a number of crystallant chemicals will absorb the emitted light and some salts are fluorescently active [primuline yellow (sodium salt), acridine yellow (hydrochloride salt) and lucifer yellow (lithium salt) to name only three].

At the Collaborative Crystallization Centre (C3; http://www.csiro.au/c3), we have recently added UV-light imaging to our automated white-light (visible) imaging. Our imaging protocol consists of 15 visible-light inspections taken over approximately ten weeks, weighted with more inspections over the first four weeks. UV-light inspections are included after the first week, after a month and at the end of the inspection period. Here, we present some of our observations from the many hundreds of samples that have been subject to UV-light illumination, with a particular focus on the results from standard crystallization test proteins.

## 2. Materials and methods   

Initial tests involved 11 test proteins that were purchased from Sigma–Aldrich (see Table 1 ▶ for a summary of concentrations and the buffers used to formulate the proteins for crystallization). These proteins were set up in a standard sparse-matrix screen (the Joint Centre for Structural Genomics screen, JCSG+, made in-house at C3) and imaged using both UV and visible light. The results of the UV imaging were compared across all of the proteins, focusing on crystallization conditions that might interfere with UV-light imaging. This was further probed by looking at four samples that had been set up in a more extensive 768-condition screen. Furthermore, as the test set of proteins included three that contained a haem group, we were interested in determining the effect of the iron-containing group on the resultant images. As a comparison, haematin (Sigma H3505) was dissolved at 2 mg ml^−1^ in dimethyl sulfoxide (DMSO) and compared with the haem-containing proteins.

### 2.1. Software and hardware   

All UV–Vis spectra were measured using an Infinite M1000Pro TECAN (Switzerland) UV–Vis spectrophotometer with a sample consisting of 200 µl solution (listed in Table 2 ▶). All of the images were collected using the C3 in-house imagers (Minstrel HT/UV, Rigaku, USA) and were displayed using the CrystalTrak application (Rigaku, USA). The Minstrel UV light is generated from an array of light-emitting diodes (LEDs) that emit light of 295 ± 5 nm. Each fluorescence image was acquired through a 1200 ms exposure to the incident UV light.

### 2.2. Screening conditions   

Nine different 96-well crystallization screens were used in this study. An exhaustive list of the contents of each screen (C3_1, C3_2, C3_3, C3_4, C3_5, C3_6, C3_7, C3_8 and JCSG+_C3) can be found on the *C*6 web tool accessed *via* the C3 home page (http://www.csiro.au/c3). The screens were prepared in-house using a TECAN (Switzerland) Evo100 liquid-handling robot; the stocks used to make the screens are also enumerated on the *C*6 web tool.

Proteins were set up in droplets consisting of 150 nl protein solution and 150 nl crystallization cocktail and were equilibrated at 293 K against a reservoir of 50 µl crystallization cocktail. All experiments were set up using a Phoenix crystallization robot (Art Robbins Industries, USA) in SD-2 plates (Molecular Dimensions, UK) and were sealed with ClearVue UV-transparent seals (MD6-01S, Molecular Dimensions, UK). 11 control proteins were set up as described above against the JCSG+_C3 screen (protein concentrations and formulations are given in Table 1 ▶). Four proteins which had been submitted to C3 for general screening against a C3 standard 768-condition (8 × 96-well) screen were also analysed. These proteins were set up in the screens C3_1 to C3_8. The proteins are listed in Table 3 ▶.

### 2.3. Protein–DNA complexes   

**Figure 1:**
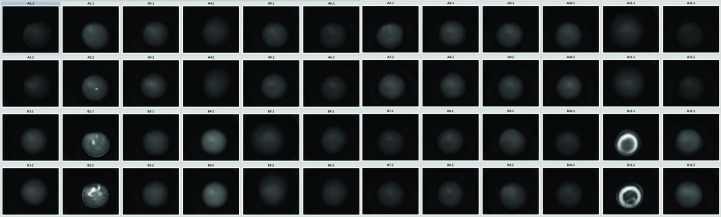
Fluorescence comparison of proteins and protein–DNA mixtures. UV images of drops A1–A12 (lysozyme alone, row 1; lysozyme and DNA, row 2) and drops B1–B12 (lysozyme alone, row 3; lysozyme and DNA, row 4); rows C–H are not shown.

To probe the effect of DNA on protein fluorescence, we compared lysozyme at 50 mg ml^−1^ in 50 m*M* Tris pH 8, 50 m*M* NaCl with the same formulation supplemented with equimolar DNA. The DNA used was an oligonucleotide with sequence 5′-GCTTCTGACAACATATGTGCG-3′ (molecular weight 6421 Da). Both the lysozyme and the lysozyme–DNA samples were set up as above against the JCSG+_C3 screen. Comparison of the resulting fluorescence intensity was performed by selecting ten pixels at random through the droplets in both the lysozyme and lysozyme–DNA samples and using the *GNU Image Manipulation Program* (*GIMP*) software package (v.2.8.2) to determine the intensity of the pixels. The intensity values for each sample were averaged and the averages of the two samples were compared (Figs. 1 ▶ and 2 ▶). This method will break down if the images of the droplets are not uniform (for example, if the droplet contains a lumpy precipitate that gives distinct patterns of light and dark in the resulting UV image), so only droplets with a reasonably smooth UV image were chosen for this analysis. We appreciate that the system described here is not a true DNA–protein complex, but contains the same components (nucleic acid and protein) as a DNA-binding protein complexed with DNA.

### 2.4. Concentration of tryptophan   

A comparison of the fluorescence for four of the test proteins (concanavalin A, proteinase K, thaumatin and thermolysin) was performed by selecting ten pixels at random through five droplets (A4, B5, D8, D11 and H2). The selection was very empirical: the pixels were selected by mouse clicking on the image ten times and checking that the pixels thus selected were unique. The intensity of the pixels was averaged per drop and then averaged per protein. The intensity averages were compared with each other with respect to the concentration of tryptophan (Table 4 ▶).

## 3. Results and discussion   

In order to illustrate the caveats associated with UV-light imaging for protein crystal identification, we initially looked at the behaviour of 11 control proteins in the sparse-matrix JCSG+ screen. Subsequently, we extended the analysis to the C3 screen (768 unique crystallization conditions) with four unrelated (noncontrol) proteins to define broad conditions that may negatively impact UV imaging. It is important to understand the two criteria that have to be met for UV fluorescence to be observed: the protein must contain chemical groups capable of absorbing energy at the excitation wavelength and the emitted energy has to avoid intermolecular and intramolecular absorbance (fluorescence quenching). The following results and discussion are based on these physical phenomena. All of our results are based on visual inspection of the crystallization drops, and the location of crystals is determined by local bright spots in the UV-fluorescent image based on the assumption that a crystal is the most concentrated form of the protein. However, a bright fluorescent spot does not immediately mean that a crystal has been located, as other phases of the protein can also lead to local high concentrations; in particular, we find that collapsed bubbles can lead to features in a UV image akin to those of crystals. Proteins may form absorption layers (PALs) at liquid–gas interfaces (Yampolskaya & Platikanov, 2006 ▶; *e.g.* an air bubble in a liquid drop); over time the bubble deflates, but the PAL remains, shrivelling in upon itself. A similar process can occur over the surface of the crystallization drop itself; as the drop equilibrates, the PAL becomes visible as a wrinkled skin. Other confounding phases can be phase separation, in which the protein concentrates into one phase, and spherulites. False positives can also be attributed to protein adsorbing onto the surface of an inorganic crystal, leading to an object that glows in the UV image but is actually salt. This last case is probably the hardest to recognize as being a false positive. The adsorbed protein on salt crystal case can often be differentiated from a protein crystal by looking at the distribution of the fluorescence: a true protein crystal will glow evenly, while a salt crystal with surface-adsorbed protein will tend to glow more patchily (see Fig. 3 ▶ for examples).

Crystal growth was observed for eight of the 11 control proteins in JCSG+ on inspection of the visible-light images and on inspection of the UV fluorescence images for proteinase K, thermolysin, apo­ferritin (spherulites, weak fluorescence), concanavalin A and thaumatin (Fig. 4 ▶). No crystals were obtained for myoglobin, haemoglobin and ferritin over the time course of the analysis. The UV–Vis spectra of the test proteins confirmed that those which displayed fluorescing crystals show absorbance between 290 and 295 nm, while those that did not absorb between 290 and 295 nm were those that did not contain tryptophan (spectra of all 11 test proteins were measured; only those of ribonuclease A and thermolysin are shown in Figs. 5 ▶ *a* and 5 ▶ *b* for brevity). As expected, the proteins without tryptophan showed no fluorescence under UV imaging. However, the presence of tryptophan does not ensure that a protein crystal will fluoresce. For example, the protein catalase contains six tryptophans per 506 amino-acid residues, but its crystals displayed no fluorescence. This is readily attributed to the haem group, which absorbs strongly in the region of 300–350 nm and prevents catalase from fluorescing even if the incident light is readily absorbed (see Figs. 5 ▶ *a*–5*d*). To confirm that this interference arises from the haem group, we measured the UV–Vis spectrum of haematin dissolved in DMSO (Fig. 5 ▶ *c*). Haematin absorbs very strongly even at 0.2 m*M* and would easily swamp the fluorescence-emitted light from any tryptophan present in the protein.

Intermolecular factors also affect fluorescence. The 768 unique conditions from the C3 screen are combinatorial mixtures of 133 distinct chemicals and the top 11 conditions that negatively influence fluorescence are listed in Table 5 ▶. This influence was deduced by visual inspection of four unrelated proteins, so that we could ensure that the quenching came from the crystallization solution and not from the protein or from interaction of the protein and crystallant. As we had observed intramolecular fluorescence quenching for proteins with a haem group, we assessed crystallant conditions containing iron (*e.g.* ferric chloride) and other divalent cations such as cobalt; we noticed that there was rarely a clear correlation between the presence of divalent cation and UV quenching and that there was often only a reduction in fluorescent intensity. We measured the UV–Vis spectra of three different iron-containing solutions and all absorbed light in the range 200–375 nm. The spectrum of a representative iron salt (0.01 *M* FeCl_3_) is shown in Fig. 5 ▶(*d*). The other conditions are more mysterious, although it seems that the combination of acetate anions and polyethylene glycol reduces the intensity of fluorescence somewhat reproducibly.

Chemical species in the protein sample itself, and not just the crystallization conditions, can modulate UV fluorescence. For example, the use of hexahistidine tags to purify proteins for crystallization is common and this process can result in the inclusion of imidazole in the final protein preparation, as it is used to elute the tagged protein from the affinity media. Willaert & Engelborghs (1991 ▶) have shown that protonated imidazole can quench tryptophan fluorescence. Eftink & Ghiron (1981 ▶) showed that other chemicals, some of which are used for phasing of diffraction images (for example, iodide), can also reduce any intrinsic tryptophan fluorescence.

All crystallization droplets containing nitrate (with and without Fe) showed completely quenched fluorescence, even if there were obvious crystals in the drop and the crystals were of protein that fluoresced under other crystallization conditions (Fig. 6 ▶). Examination of the UV–Vis spectrum for nitrate shows that this anion absorbs in the region 300–350 nm and thus absorbs the emitted light (Fig. 5 ▶ *e*), illustrating that intermolecular quenching is important to consider for UV-fluorescent imaging of protein crystals. A grid-based optimization screen was created for an in-house project that contained polyethylene glycol in all conditions, along with HEPES or Tris buffers, and one of four magnesium salts with acetate, chloride, formate or nitrate counter anions. The fluorescence observed in this screen clearly confirms that the nitrate anion attenuates fluorescence (Fig. 6 ▶). A spectrum of sodium nitrate (Fig. 5 ▶ *e*) shows that there is absorbance from this anion in the range 280–330 nm.

To test the hypothesis that the brightness of UV fluorescence is independent of the sequence of the protein (outside the absolute requirement that at least one tryptophan is present), we compared the fluorescence of similar drops set up with different proteins. The brightness of the fluorescence from five drops of four of the control proteins (JCSG+ conditions A4, B5, D8, D11 and H2) was measured by looking at ten individual pixels chosen at random throughout the drop. This method requires that the brightness is even over the drop, and only four of the test proteins had crystallization conditions in common which had the required visual smoothness. The average brightness of these four proteins over five drops was calculated and compared with the concentration of tryptophan in the protein sample (Table 4 ▶). In this study, we used the control proteins at concentrations known to be suitable for crystallogenesis; we were not trying to match the protein concentration (or even the tryptophan concentration) between the test proteins. We confirmed that there is no direct correlation between the concentration of tryptophan in the drop and the brightness of the emitted light, reaffirming that the tryptophan fluorescence differs depending on the position (exposed or buried) of the side chain in the protein (Gill, 2010 ▶).

**Figure 2:**
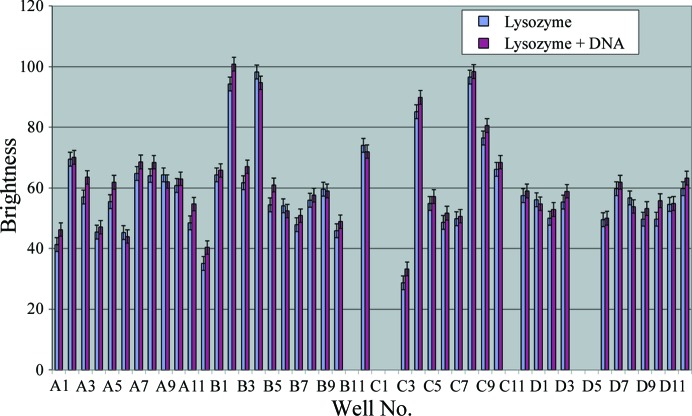
Intensity of the fluorescence for protein and protein–DNA mixture, where 0 is black and 255 is white (average of ten randomly chosen pixels from within the drop), over 80 homogeneous conditions of JCSG+. Missing values were not measured owing to an uneven brightness within the drop, for example B11, visible in Fig. 1 ▶. The average is 58.76, with a standard deviation of 14.77.

**Figure 3:**
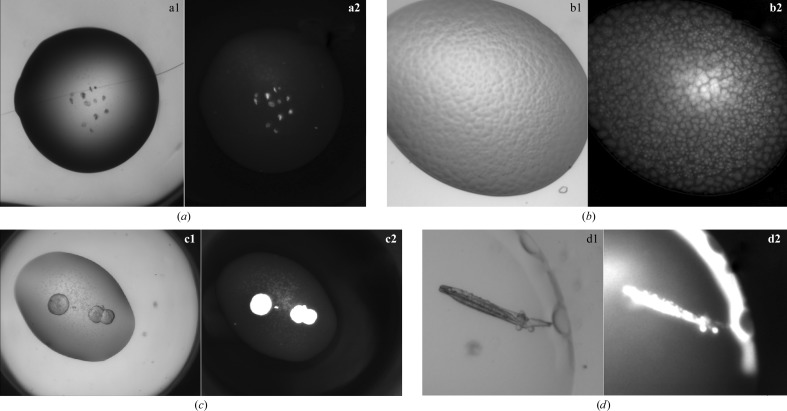
False positives under UV light. (*a*) Visible-light (a1) and UV-light (a2) pictures of burst bubbles in a crystallization experiment; (*b*) visible-light (b1) and UV-light (b2) pictures of a phase separation; (*c*) visible-light (c1) and UV-light (c2) pictures of spherulites; (*d*) visible-light (d1) and UV-light (d2) pictures of a salt crystal with adsorbed protein (on the surface of the salt crystal).

**Figure 4:**
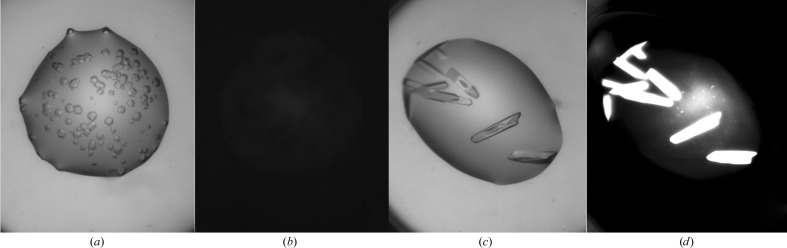
False-negative and true-positive trials under UV light. (*a*, *b*) Insulin crystals with the corresponding UV image. (*c*, *d*) Concanavalin A crystals with the corresponding UV image.

**Figure 5:**
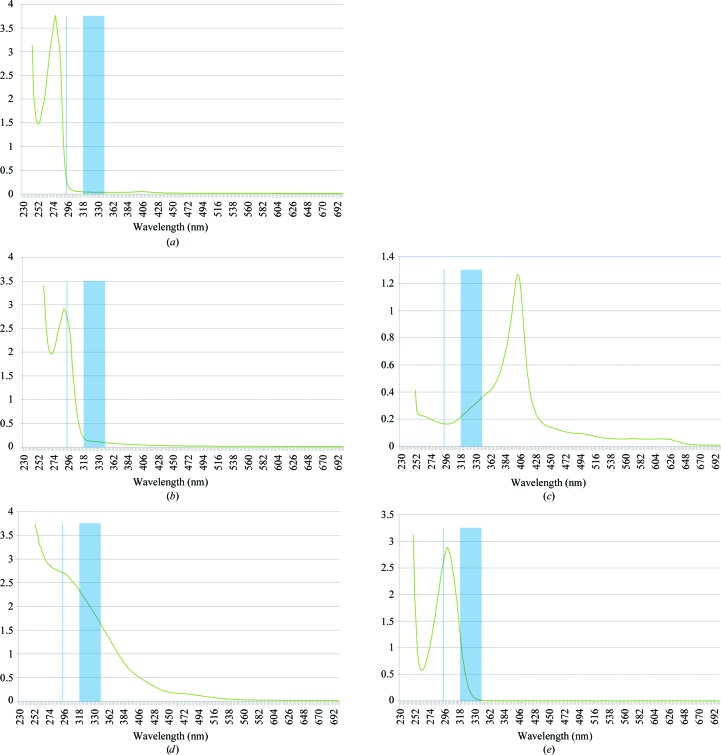
UV–Vis spectra of different proteins and chemical solutions. (*a*) Ribonuclease A (at 30 mg ml^−1^) is non-absorbing at 295 nm. (*b*) Thermolysin (at 2.5 mg ml^−1^) absorbs at 295 nm. (*c*) Haematin (at 0.21 m*M*) absorbs at both 295 and 320–350 nm. (*d*) FeCl_3_ absorbs at both 295 and 320–350 nm. (*e*) Sodium nitrate absorbs at 295 and 320–330 nm. For all graphs, the vertical line is at 295 nm and the vertical band is between 320 and 350 nm.

**Figure 6:**
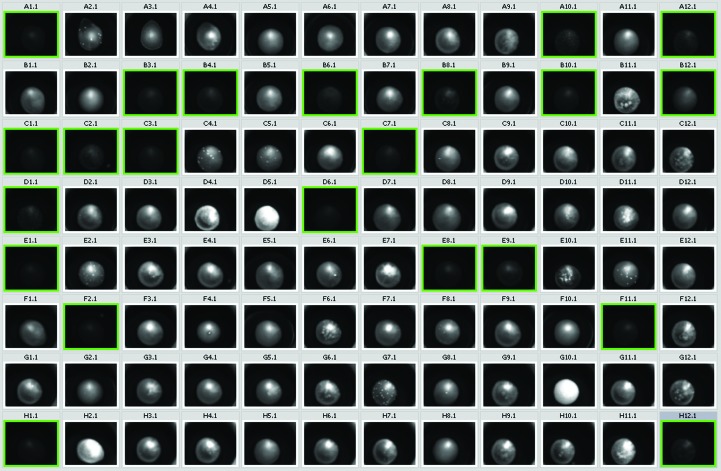
UV shot of a protein plate in which conditions containing nitrate are highlighted in green.

To check if the presence of DNA in the protein solution has any effect on the fluorescence (quenching or enhancement), we set up one plate of 2 × 96 wells with reservoir in which 96 wells were under JCSG+ conditions with lysozyme and 96 wells were under the same conditions with lysozyme and DNA in an equimolar ratio (this ratio was chosen as it is the ratio usually used for protein–DNA experiments; Hollis, 2007 ▶). Within the error margins, there was no difference in brightness between the drops containing only lysozyme and the drops containing lysozyme and DNA. Only drops A1–D12 are shown in Fig. 2 ▶; similar results were obtained for drops E1–H12. The presence of DNA does not appear to affect the fluorescence.

## 4. Conclusion   

**Figure 7:**
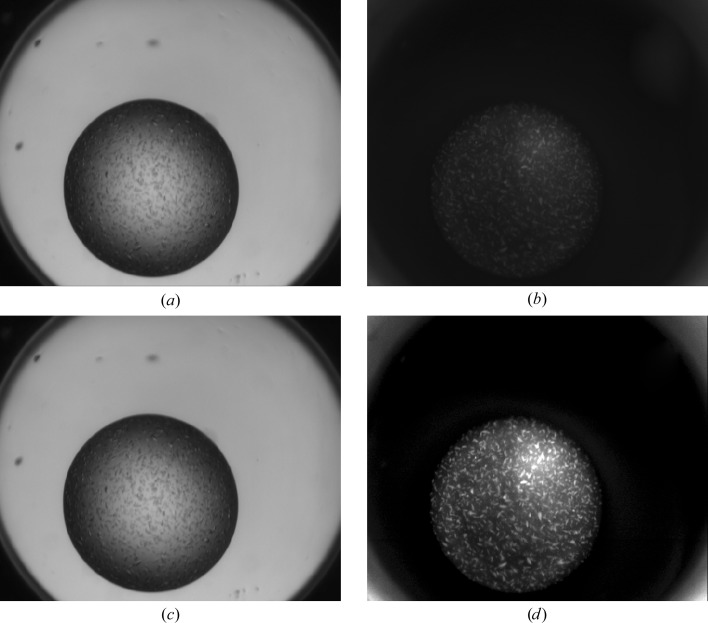
A UV-light display increase allows crystals to be observed even when quenching by the cocktail occurs (nitrate). (*a*, *c*) Drop with protein crystals under visible light. (*b*) The same drop under normal UV-light display. (*d*) The same drop under enhanced UV-light display.

This work reinforces the observation that UV imaging is based on tryptophan fluorescence and that this technique is inappropriate in the absence of tryptophan in the protein. A similar lack of fluorescence is often observed for haem-containing proteins, which are self-quenching. The best way to determine whether crystals will fluoresce under UV is to measure a spectrum of the protein solution between 290 and 350 nm. If the solution absorbs between 320 and 350 nm, no fluorescence will be observed as it will be reabsorbed by the solution. The addition of nucleic acid to the protein solution does not add any further caveats to this general conclusion. Some chemical conditions, particularly those which contain nitrates and/or some metals (cobalt, iron), have a negative effect on either absorbed or emitted UV light. This results in a dimming or quenching of any protein fluorescence and may mask the appearance of crystals in the UV image. Most of the time, when quenching occurs owing to the crystallization cocktail it only decreases the intensity of the fluorescence, and increasing the gain of the monitor used to view the UV image may allow any crystals to still be observed (Fig. 7 ▶). Brightly glowing objects in a UV image are often crystals, but care must be taken to compare the UV image with a similar white-light image in order to identify spurious glowing objects, in particular collapsed bubbles.

## Figures and Tables

**Table 1:** The 11 test proteins with their formulation buffers and concentrations EDTA, ethylenediaminetetraacetic acid; PMSF, phenylmethanesulfonyl fluoride.

Protein	Concentration (mgml^1^)	Buffer	No. of tryptophans	No. of residues	Comments
Catalase	30	25m*M* HEPES pH 7	6	506	Haem protein
Haemoglobin	40	100m*M* NaH_2_PO_4_ pH 6.5	1	141	Haem protein
Myoglobin	20	H_2_O	2	153	Haem protein
Ferritin	20	50m*M* NaCl	1	183	Iron-containing
Ribonuclease A	30	100m*M* sodium acetate pH 6	0	124	Tryptophan-free
Insulin	20	20m*M* Na_2_HPO_4_, 10m*M* Na_3_EDTA	0	21	Tryptophan-free
Proteinase K	20	25m*M* Tris pH 7.5, 1m*M* PMSF	2	279	
Thaumatin	50	100m*M* sodium/potassium tartrate	3	207	
Thermolysin	25	50m*M* NaOH	3	316	
Apoferritin	20	H_2_O	1	174	
Concanavalin A	50	H_2_O	4	237	

**Table 2:** The test proteins and solutions used for the UVVis spectra with their buffers and concentrations DMSO, dimethyl sulfoxide.

Protein or solution	Concentration (mgml^1^)	Buffer
Catalase	7.5	25m*M* HEPES pH 7
Haemoglobin	1.25	100m*M* Na/H_2_PO_4_ pH 6.5
Myoglobin	1.25	H_2_O
Ferritin	0.625	50m*M* NaCl
Ribonuclease A	15	100m*M* sodim acetate pH 6
Insulin	10	20 m*M* Na_2_/HPO_4_ 10m*M* Na_3_EDTA
Proteinase K	10	25m*M* Tris pH 7.5, 1m*M* PMSF
Thaumatin	6.25	100m*M* sodium/potassium tartrate
Thermolysin	3.125	50m*M* NaOH
Apoferritin	5	H_2_O
Concanavalin A	6.25	H_2_O
Haematin	0.158	DMSO
FeCl_3_	1.6	H_2_O
Sodium nitrate	60	H_2_O

**Table 3:** The four proteins used in the 768-condition screen TBS, Tris-buffered saline. TCEP, tris(2-carboxyethyl)phosphine. The four proteins used inthis study were samples from clients of C3 and were not part of the test set of commercially available proteins.

Protein	No. of tryptophans	Concentration (mgml^1^)	Buffer	Size (kDa)
*A*	4	10	TBS pH 8	20.3
*B*	3	10	TBS pH 8	20.5
*C*	6	10	TBS pH 8	42.7
*D*	2	5.3	200m*M* NaCl, 20m*M* Tris pH 8, 10% glycerol, 0.5m*M* TCEP	32.9

**Table 4:** The four proteins tested for their comparative fluorescence along with their tryptophan concentration

Protein	Tryptophan concentration (gml^1^)	Brightness
Concanavalin A	15.97	208.72
Proteinase K	2.83	152.44
Thaumatin	13.80	99.33
Thermolysin	4.46	171.82

**Table 5:** Cocktails found in the C3 screen (768 conditions) which showed little fluorescence, as observed for four different proteins in this screen Conditions that contained nitrate or cobalt were excluded from the analysis, as these conditions were already known to eclipse fluorescence. CTAB, cetyltrimethylammonium bromide.

Reservoir solution	Screen	Well
10% Jeffamine M-600 pH 7, 100m*M* trisodium citratecitric acid, 10m*M* ferric chloride	C3_1	A1
12% PEG monomethyl ether 750, 100m*M* sodium acetateacetic acid pH 5	C3_1	H5
10% PEG 3000, 20m*M* zinc acetate, 100m*M* sodium acetateacetic acid pH 5	C3_2	E6
15% PEG 4000, 50m*M* sodium cacodylate pH 6, 200m*M* potassium thiocyanate	C3_3	F4
2.5% 1-octanol, 15% polypropylene glycol P400	C3_4	H8
1*M* sodium acetate, 100m*M* imidazole pH 6.5	C3_5	A1
500m*M* sodium chloride, 10m*M* CTAB, 10m*M* magnesium chloride	C3_5	B6
1*M* imidazole pH 6.5	C3_5	H6
0.8% *n*-octyl--D-glucopyranoside, 2*M* ammonium sulfate, 20% ethylene glycol	C3_6	C11
35% *tert*-butanol, 10m*M* trisodium citratecitric acid pH 5.5	C3_8	H11
2*M* sodium chloride, 40% trichloroacetic acid	C3_8	H12
